# Triple Intussusception in an Adult—A Rare Presentation of Adenocarcinoma Ileum

**DOI:** 10.1055/s-0041-1733834

**Published:** 2021-10-05

**Authors:** Kirankumar P. Jadhav, Gayathri Krishnan

**Affiliations:** 1 Department of Surgery, B. J. Government Medical College and Sassoon General Hospital, Pune, Maharashtra, India

**Keywords:** adult intussusceptions, adenocarcinoma ileum, multiple intussusceptions

## Abstract

Intestinal intussusception is uncommon in adults. It occurs more often in the small intestine than in the colon. In adults, when small bowel intussusception occurs, it can be due to a malignant lead point. Malignant etiology is most frequently due to diffuse metastatic disease. We present a rare case of an 18-year-old woman who was diagnosed with jejunojejunal, jejunoileal, and colocolic intussusceptions. She presented with vomiting, abdominal pain, and passage of semisolid stools for 5 days. During emergency exploratory laparotomy, multiple polyps were found in the jejunum, ileum, and sigmoid. Jejunotomy and sigmoidotomy were done to remove the respective polyps. The ileal polyp showed hemorrhagic changes; hence, an intraoperative decision was taken to proceed with resection and anastomosis. On histopathological examination, the resected ileal part showed moderately differentiated adenocarcinoma (grade 2) arising from an adenomatous polyp, while the jejunal polyp and sigmoid polyp were adenomatous polyps with low-grade dysplasia. Patient received six cycles of adjuvant chemotherapy consisting of capecitabine and oxaliplatin (CAPEOX regimen). After 2 years, she is symptom free with a normal colonoscopy. The treatment of intussusception in adults typically involves surgery, often with bowel resection as there is always a pathologic leading cause which may be malignant, like in our case.

Intussusception involves the telescoping of a segment of bowel into an adjacent segment. This leads to complications such as obstruction, inflammation, and possible ischemia. Intussusception is the leading cause of intestinal obstruction in children. However, adult intussusception is rare, accounting for less than 5% of all bowel obstructions and 5% of all intussusceptions. 1 2 Simultaneous occurrence of multiple intussusceptions is a rare condition, exact incidence of which is still unavailable. 3

Intussusceptions in children are typically primary or idiopathic. In contrast, most adult intussusceptions have a demonstrable etiology in 70 to 90% of the cases. 4 Sixty-six per cent of colonic intussusceptions and 30% of cases in the small intestine have malignant neoplasms as their lead point. Adenocarcinoma is the most common malignant lead point in the colon, whereas metastasis is the most common in the small intestine. 5 6

Small bowel neoplasms are exceedingly rare, constituting only 5% of all gastrointestinal neoplasms and only 1 to 2% of all gastrointestinal malignant tumors, despite the fact that they comprise 90% of the length of the alimentary tract. 7 8 Malignant neoplasms of the small bowel are primarily of two etiologies: neuroendocrine tumors which account for ∼40% of cases and adenocarcinoma which account for another 40%. The remaining 20 to 25% comprise gastrointestinal stromal tumors, sarcomas, and lymphomas. 9 In descending order of frequency, small bowel adenocarcinoma may occur within the duodenum (48.4%), jejunum (32.5%), or ileum (19.2%). 10

We report a rare case of multiple simultaneous small bowel intussusceptions secondary to adenocarcinoma of the ileum in an 18-year-old woman. The statistics mentioned earlier emphasizes the rarity in reporting of these two conditions occurring together.

## Case Report

An 18-year-old woman presented to the OPD with complaints of vomiting, abdominal pain, and passage of semisolid stools for 5 days. There was no history of fever, hematemesis, or malena. She did not have similar episodes in the past. On examination, she was vitally stable. She had diffuse tenderness and distension on per abdominal examination. Rest of abdominal examination was unremarkable. Per rectal examination was normal.

**Fig. 1:**
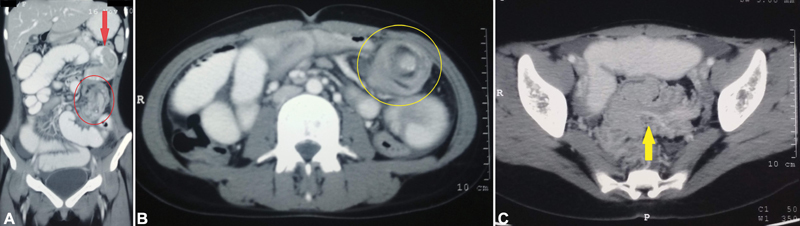
Contrast-enhanced computed tomography of the abdomen showing ( **A** ) jejunojejunal intussusception (marked with red arrow) and ileoileal intussusception (circled with red) in coronal section, ( **B** ) ileoileal intussusception (circled with yellow) in sagittal section, and ( **C** ) colocolic intussusception involving sigmoid (yellow arrow) in sagittal section.

On investigating further, plain X-ray of the abdomen showed no abnormalities. Ultrasonography of abdomen and pelvis revealed: ileocolic intussusception in the right iliac fossa with ileum extending into ascending colon up to right half of transverse colon; ileoileal intussusception in left iliac fossa for a length of 8 cm; multiple enlarged mesenteric lymph nodes of average size 10 mm; and mild ascites. A contrast-enhanced computed tomography (CECT) of the whole abdomen confirmed the diagnosis demonstrating three regions of intussusceptions, namely, jejunojejunal, jejunoileal, and colocolic intussusceptions and irregular wall thickening involving the sigmoid colon ( Fig. 1 ).

**Fig. 2:**
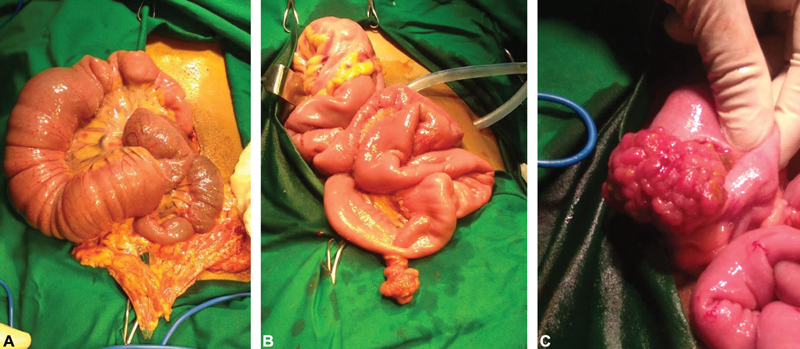
Intraoperative findings ( **A** ) ileoileal intussusception, ( **B** ) jejunal polyp, and ( **C** ) sigmoid polyp.

After obtaining consent, patient was taken up for emergency exploratory laparotomy. All three intussusceptions were reduced without any complications. Bowel wall was mildly edematous without any obvious signs of ischemia/necrosis. Multiple polyps in the jejunum, ileum, and sigmoid colon were found to be lead points ( Fig. 2 ). Jejunotomy and sigmoidotomy were done to remove the respective polyps. The ileal polyp was hemorrhagic in appearance, which was suspicious of malignancy. Since frozen section was not available at the time of surgery, an intraoperative decision was made to proceed with ileal resection with 5 cm margin proximal and distal to polyp. The specimens were sent for histopathological examination. Rest of the abdomen was examined and found to be normal. The patient tolerated the surgery well. She made an uneventful recovery. Patient was discharged on postoperative day 5.

**Fig. 3:**
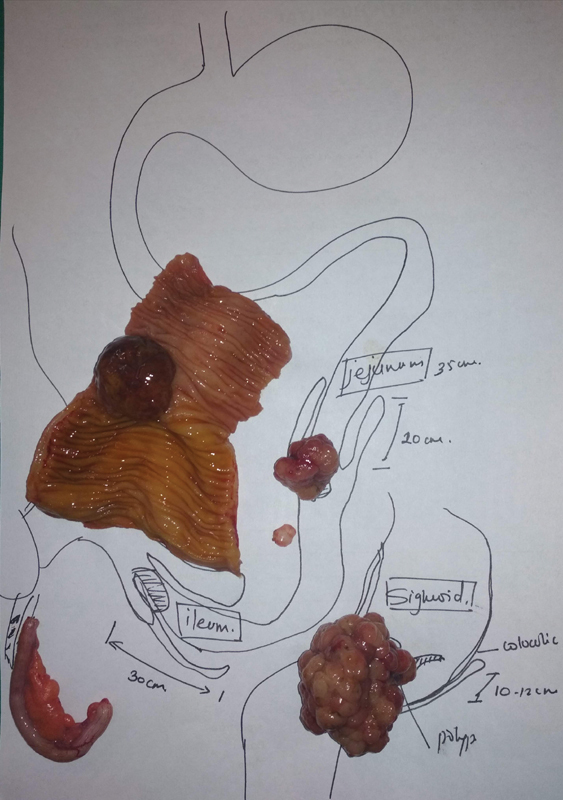
Orientation of the resected specimen.

Histopathological report revealed: resected ileal segment—moderately differentiated adenocarcinoma (grade II) arising on the background of adenomatous polyp; jejunal polyp—adenomatous polyp with low-grade dysplasia; sigmoid polyp—adenomatous polyp with low-grade dysplasia; and appendix—chronic appendicitis (no granulomas/malignancy) ( Fig. 3 ).

On follow-up, patient was started on CAPEOX regimen (tablet capecitabine 500 mg 2–0–2 × 14 days injection oxaliplatin 180 mg), 21-day cycles for 6 cycles. Patient responded well to the regimen. Positron emission tomography–CT done 3 months postoperatively showed fluorodeoxyglucose avid small benign polyp in anterior wall of rectum for which polypectomy was done. Patient is symptom free and doing well till date with regular follow-up of 2 years.

## Discussion

Intussusception is defined as the invagination of one segment of the bowel into an immediately adjacent segment. It can occur anywhere in the bowel. Intussusception in children usually occurs between 6 and 18 months of age. The incidence of intussusception declines with age accounting for only 1 to 5% of bowel obstructions in adults. 1 2 11

In the pediatric population, intussusceptions are typically primary or idiopathic with only 10% of cases having an identifiable precipitating lesion. In contrast, 70 to 90% of the cases of adult intussusceptions are caused by a structural lesion/lead point pathology which can be intraluminal, mural, or extramural. 1 2

A lead point intussusception involving the small bowel is generally due to a benign condition. 12 Nonmalignant etiology includes benign tumors, adhesions, lymphoid hyperplasia, cystic fibrosis, scleroderma, celiac disease, inflammatory bowel disease, appendicitis, pancreatitis, and rectal foreign bodies. Sixteen per cent of small bowel and 5% of large bowel intussusceptions are idiopathic. 6

Sixty-six per cent of colonic intussusceptions and 30% of intussusceptions in the small intestine occur due to malignant lead points. The most common malignant lead point in colon is adenocarcinoma and in the small intestine, it is diffuse secondary metastasis. 6 7

Our patient is an 18-year-old woman, who presented to us with multiple intussusceptions of small bowel due to adenocarcinoma of the ileum, both of which are rare entities.

Adults rarely present with the classic triad of abdominal pain, bloody currant jelly stools, and palpable tender abdominal mass seen in children. They may present as acute intestinal obstruction/perforation or more commonly chronic intermittent cramping abdominal pain associated with nonspecific signs of bowel obstruction. 2 The nonspecific nature of these findings can result in a broad differential diagnosis.

Preoperative diagnosis ranges from 30 to 70%. 4 13 Evaluation often starts with X-ray of the abdomen which may reveal signs of intestinal obstruction or perforation but it lacks sensitivity and specificity for diagnosing intussusception. 14

Ultrasound is both diagnostic and therapeutic with 100% sensitivity and specificity in children. 15 Nonoperative reduction of the intussusceptum by ultrasound-guided or fluoroscopic pneumatic or hydrostatic enema is successful in 85 to 90% of pediatric cases. 16 However, ultrasound tends to be less accurate in adults but may still reveal classic features. In our patient, two out of three intussusceptions could be detected by ultrasound, but etiology could not be identified.

Abdominal CECT represents the most accurate diagnostic modality with the possibility of demonstrating lead points, vascular compromise, and possible associated complications such as intestinal obstruction or local spread. 17 Classic radiological findings include “target,” “bulls-eye,” or sausage-shaped lesions as a concentric hyperdense double ring, features owing to the anatomic configuration of the outer intussuscipiens and the central intussusceptum creating a bowel-within-bowel appearance. 13 Multiple intussusceptions and irregular wall thickening involving the sigmoid colon could be identified on CECT in our patient.

The approach to management of intussusception is different in pediatric and adult intussusceptions. While in children, treatment is primarily based on radiological reduction, surgical resection is recommended in cases of adult intussusception because it is often associated with a lead point which can be malignant.

A selective approach to bowel resection is recommended when patient requires operative management, depending on the age, pathology, and location of lead point. Bowel resection following the appropriate oncologic principles is recommended given the relatively high incidence of malignancy in adults. 6 Curative resection is the treatment of choice and a chance for long-term survival. Lymphovascular invasion and positive surgical margins are predictors of locoregional recurrence and poor outcome. 18 In our patient, multiple dysplastic polyps could be found which warranted resection of bowel segment.

The suggested first-line adjuvant chemotherapy for adenocarcinoma of ileum is a combination regimen of 5-fluorouracil infusion or oral capecitabine with oxaliplatin. 8 Other agents such as irinotecan and gemcitabine have also been tried. The use of biological agents and targeted therapy in patients with this rare tumor is under trial. 9 Our patient was given six cycles of CAPEOX regimen. She is recurrence free till date.

## Conclusion

Adult intussusception is a rare entity and challenging to diagnose due to nonspecific symptoms with a vast differential diagnosis. Small bowel tumors are a rare but possible etiology of adult intussusception. Surgical exploration is the only way of making and confirming the diagnosis of small bowel adenocarcinoma despite the availability of sophisticated imaging modalities.
